# Plastic response of the oyster *Ostrea chilensis* to temperature and *p*CO_2_ within the present natural range of variability

**DOI:** 10.1371/journal.pone.0234994

**Published:** 2020-06-29

**Authors:** Jorge M. Navarro, Paola Villanueva, Natalia Rocha, Rodrigo Torres, Oscar R. Chaparro, Samanta Benítez, Paola V. Andrade-Villagrán, Emilio Alarcón

**Affiliations:** 1 Facultad de Ciencias, Instituto Ciencias Marinas y Limnológicas, Universidad Austral de Chile, Valdivia, Chile; 2 Centro Fondap de Investigación Dinámica de Ecosistemas Marinos de Altas Latitudes (IDEAL), Universidad Austral de Chile, Valdivia, Chile; 3 Centro de Investigación en Ecosistemas de la Patagonia (CIEP), Coyhaique, Chile; 4 Programa de Doctorado en Biología Marina, Facultad de Ciencias, Universidad Austral de Chile, Valdivia, Chile; 5 Facultad de Ciencias, Centro de Investigación e Innovación para el Cambio Climático (CiiCC), Universidad Santo Tomás, Santiago, Chile; 6 Facultad de Ciencias, Centro de Investigación en Biodiversidad y Ambientes sustentables (CIBAS), Universidad Católica de la Santísima Concepción, Concepción, Chile; Bigelow Laboratory for Ocean Sciences, UNITED STATES

## Abstract

Estuaries are characterized by high fluctuation of their environmental conditions. Environmental parameters measured show that the seawater properties of the Quempillén estuary (i.e. temperature, salinity, *p*CO_2_, pH and ΩCaCO_3_) were highly fluctuating and related with season and tide. We test the effects of increasing temperature and *p*CO_2_ in the seawater on the physiological energetics of the bivalve *Ostrea chilensis*. Juvenile oysters were exposed to an orthogonal combination of three temperatures (10, 15, and 20°C) and two *p*CO_2_ levels (~400 and ~1000 μatm) for a period of 60 days to evaluate the temporal effect (i.e. 10, 20, 30, 60 days) on the physiological rates of the oysters. Results indicated a significant effect of temperature and time of exposure on the clearance rate, while *p*CO_2_ and the interaction between *p*CO_2_ and the other factors studied did not show significant effects. Significant effects of temperature and time of exposure were also observed on the absorption rate, but not the *p*CO_2_ nor its interaction with other factors studied. Oxygen consumption was significantly affected by *p*CO_2_, temperature and time. Scope for growth was only significantly affected by time; despite this, the highest values were observed for individuals subject to to 20°C and to ~1000 μatm *p*CO_2_. In this study, *Ostrea chilensis* showed high phenotypic plasticity to respond to the high levels of temperature and *p*CO_2_ experienced in its habitat as no negative physiological effects were observed. Thus, the highly variable conditions of this organism’s environment could select for individuals that are more resistant to future scenarios of climate change, mainly to warming and acidification.

## Introduction

Increased *p*CO_2_ in the atmosphere alters the temperature and pH of marine habitats affecting marine life at global scales [1]. CO_2_ is not an isolated environmental driver of climate change, rather its effects on life are tied to other environmental shifts such as ocean warming [2,3]. The *p*CO_2_ can affect marine organisms in two ways. First, decreases in calcium carbonate saturation can affect shell dissolution. Although mollusk shell biomineralization is mainly biologically-controlled, environmental conditions, such as calcium carbonate/aragonite chemistry, can affect this process by modifying the shell morphology, mineralogy, structural organization and the amount and composition of shell organic components (e.g., periostracum and shell organic matrix) [4].

Secondly, high concentrations of CO_2_ can alter the acid-base physiology of marine animals [5], trigger metabolic depression [6] and can also increase basal metabolic costs and reduce energy available for growth and reproduction [7]. Thus, the addition of anthropogenic CO_2_ to the ocean is seen as a major threat to marine calcifying invertebrates. Because many marine organisms live close to their thermal compensatory capacity [8], increases in temperature is expected to impact all physiological processes related with energy acquisition and energy expenditure (i.e. energy ingested and absorbed, oxygen uptake and the index scope for growth) survival, and many ecological interactions [9]. The interaction between elevated seawater *p*CO_2_ and high temperature can reduce the thermal tolerance window of an organism exposed to high CO_2_ levels [10]. However, warming may diminish the negative impacts of acidification on the calcification of juveniles and adults [11]. Increased temperature stimulates development, whereas hypercapnia can depress developmental processes. Increased CO_2_ negatively affects reproduction [12,13], calcification [14–16], and the physiology of marine invertebrates [17–19]. The combined effects of ocean acidification and other environmental variables remain poorly understood, and in order to predict organisms' responses to a wide range of environmental variability, experiments involving more than one environmental factor are necessary at different time scales and at all levels of biological organization. There is evidence that many environmental stressors can act in synergistic, additive, and/or antagonistic ways to affect various physiological processes of marine organisms [20]. Some organisms exposed to high levels of CO_2_ have decreased thermal tolerance [21], yet increased temperature can counteract the effects of low pH [11–22].

Coastal zones and estuaries experience more accented changes in water temperature and pH compared to those occurring in the open ocean [23]. Estuarine habitats, which are hotspots for biological diversity, are likely to be strongly affected by increases in atmospheric CO_2_ due the lower buffering capacity of these ecosystems [24]. Acidification can exacerbate the environmental variability of these habitats that already experience significantly higher concentrations of CO_2_ [25]. Thus, organisms inhabiting estuarine environments are subjected to periods of stress due to large fluctuations of the environmental conditions (e.g. temperature, salinity, *p*CO_2_/pH).

The Chilean oyster *Ostrea chilensis* (Philippi 1845) inhabits coastal and estuarine areas of the mid to high latitudes of southern Chile. In these areas, this species is extracted from natural beds by artisanal fishermen who cultivate this resource at a small-scales. *Ostrea chilensis* is a study model with particular biological characteristics, since it presents a long incubation period (eight weeks) and a short pelagic larval duration (e.g. several hours; [26,27]. *Ostrea chilensis* used in this study inhabit at the Quempillén estuary, characterized by its high environmental variability throughout the year [28]. Thus, these organisms are expected to have a great physiological plasticity to live in this environment. However, the extreme environmental conditions occur only in short periods of time, where in addition to a plastic response the bivalves may be able to close their valves and isolate themselves, until suitable conditions occur again.

In view of the ecological and economical importance of the oyster *Ostrea chilensis*, a detailed analysis of the energy budget was performed to understand the effects of seasonal environmental changes on the degree of physiological plasticity to its environment. For this purpose, processes related with energy gain (clearance and ingestion rates, absorption) and energy expenditure (oxygen uptake) were measured to calculate the scope for growth, index that represents an integrated response of the whole organism.

The objective of the present study was to investigate the physiological plasticity of *Ostrea chilensis* in response to temperature and *p*CO_2_ within the current natural range of variability of the Quempillén estuary, allowing to make better predictions about the tolerance range of sensitivity to future climate change conditions predicted.

## Material and methods

### Animal collection and experimental design

**Fig 1:**
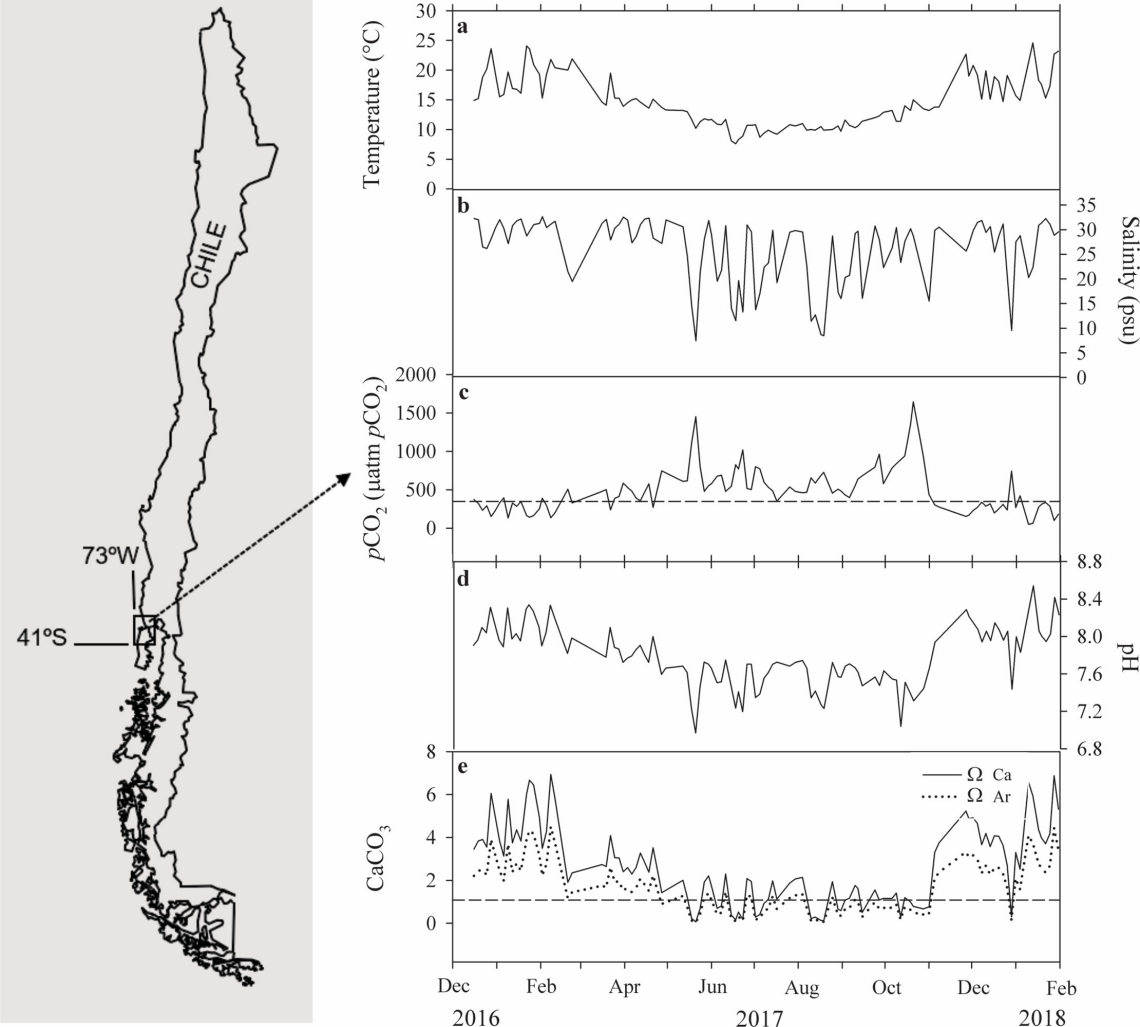
Annual variation of environmental parameters at the Quempillén estuary, Southern Chile. Time series of temperature (a), salinity (b), *p*CO_2_ (c), pH (d) and ΩCaCO_3_ (e). Map taken from Lagos et al. (2016).

Experimental juvenile individuals of *Ostrea chilensis* with shell length ranging from 2.8–3.2 cm were collected from the natural bank of the Quempillén estuary (41° 52' S, 73° 46' W), Chiloé Island, southern Chile in the winter season (July 2017). No specific permissions were required for this location. In Chile there is free access to the coast with the public coastal footpath around nearly the whole country. Only for the case of protected areas and National Parks it is necessary to ask special permission. Our field studies do not involve endangered or protected species. After collection, 60 experimental oysters were transported (5 h) in chill conditions (10 ºC) to the Universidad Austral de Chile, Valdivia, acclimated for two weeks in 20 L aquaria with seawater at the annual mean temperature (13 ± 1°C) and salinity (27) of the estuary, with permanent aeration, and water change every two days. After this period of acclimation, 2 oysters per replicate were marked using bee taggs (five replicates and 6 treatments) and gradually transferred to the mesocosm system located at the Calfuco, Coastal Laboratory, Universidad Austral de Chile. In the mesocosm, the temperature increased/decreased 1°C daily until the experimental temperatures of 10, 15 and 20°C were reached under two conditions of *p*CO_2_, the control ~400 μatm, which represents the lower values in their habitat and 1000 μatm, which represents the higher values measured, with the exception of two extreme values that were observed only twice in the estuary during the year (see Fig 1). The experimental oysters were randomly assigned to six different treatments (i.e. ~400 μatm 10 ºC; ~400 μatm 15 ºC, ~400 μatm 20 ºC; ~1000 μatm 10 ºC; ~1000 μatm 15 ºC and ~1000 μatm 20 ºC). These temperatures are within the natural thermal range experienced by *O*. *chilensis* throughout the year at the bottom of the estuary where they live. The experimental oysters were fed continuously with the microalga *Isochrysis galbana* using two multichannel peristaltic pumps (Cole-Parmer Masterflex 7524) that supplied a daily amount of food equivalent to 4% of the dry weight of the oysters. The food concentration corresponded to ca. 2.0 mg L^-1^ dry weight, which is within the natural range found in the fjords of southern Chile [29].

To simulate the current conditions of ocean acidification, the mesocosm system described by Torres et al. [30] was modified, and now an open water flow system through experimental aquariums was included. Six 250 L tanks were filled each day with filtered sea water (1 μm) from the water collection system of the Calfuco laboratory. The filtered seawater was diluted to a salinity of 27 and equilibrated with air+CO_2_ mixtures; three of the tanks were equilibrated to low *p*CO_2_ (~ 400 μatm) and the other three were equilibrated to high *p*CO_2_ (~ 1000 μatm), using mass flow controllers. The two contrasting *p*CO_2_ levels were achieved by bubbling air or by bubbling an air+CO_2_ mixture. Water was pumped from the tanks into 20 L drums immersed in temperature-controlled trays (using 3 thermoregulatory devices; Alpha Lauda R8).

Thirty 5 L experimental aquaria were installed; five aquaria (replicates) were designated to each treatment (i.e. each combination temperature/*p*CO_2_ levels). The flow of water through each experimental aquarium was maintained constant using an open circulation system. The flow rate was set to 1.8 L h^-1^, which allowed the volume of water in the aquaria to be renewed approximately nine times per day in minimize the accumulation of metabolites, alkalinity deficit and oxygen deficit associated to calcification and respiration. A slight bubbling of air was maintained in each experimental aquarium in order to ensure oxygen saturation (i.e. atmospheric *p*CO_2_, i.e. near ~400 μatm), however by doing so high *p*CO_2_ (i.e. nominally ~1000 μatm) aquaria drop slightly its *p*CO_2_ relative to the nominal value.

Since CO_2_ equilibration was achieved before temperature equilibration, the changes in CO_2_ solubility changed slightly the final omega, pH and *p*CO_2_ depending of the temperature gradient between 250 L tanks (no temperature controlled) and 20 L drums, likely involving departures of the order 3%, 1% and 19%, respectively, from the target. Final *p*CO_2_ and other temperature dependent carbonate system parameters, i.e. calculated from measured pH and A_T_ (Table 1), include all sources of variability, including the adjustment of *p*CO_2_ previous to temperature.

**Table 1:** Carbonate system parameters during the experiment conducted with juvenile *Ostrea chilensis*.

Carbonate system parameters	Low *p*CO_2_			High *p*CO_2_		
	10°C	15°C	20°C	10°C	15°C	20°C
pH at 25.0°C	7.76 ± 0.01	7.75 ± 0.01	7.73 ± 0.02	7.53 ± 0.04	7.55 ± 0.04	7.58 ± 0.02
pH _in situ_ (Total scale)	7.98 ± 0.01	7.90 ± 0.01	7.80 ± 0.02	7.73 ± 0.05	7.68 ± 0.05	7.65 ± 0.05
Salinity (PSU)	27.60 ± 0.09	27.55 ± 0.08	27.64 ± 0.03	27.44 ± 0.09	27.62 ± 0.05	27.77 ± 0.12
A_T_ (μmol kg^-1^)	1838.80 ± 14.07	1807.45 ± 16.32	1775.89 ± 20.85	1823.32 ± 8.98	1784.53 ± 39.65	1774.34 ± 37.39
*p*CO_2_ (μatm)	396.34 ± 9.74	494.69 ± 13.88	623.17 ± 27.93	767.43 ± 90.31	864.05 ± 101.22	921.11 ± 34.91
[HCO_3_^-^] (μmol kg^-1^)	1635.73 ± 13.10	1609.30 ± 13.38	1584.24 ± 18.74	1703.04 ± 17.51	1657.13 ± 38.81	1636.11 ± 33.14
[CO_3_^2-^] (μmol kg^-1^)	80.24 ± 1.68	78.63 ± 2.25	76.22 ± 2.77	47.43 ± 4.87	50.37 ± 5.24	54.96 ± 2.74
Ω_calcite_	1.99 ± 0.04	1.96 ± 0.06	1.92 ± 0.07	1.18 ± 0.12	1.26 ± 0.13	1.38 ± 0.07
Ω_aragonite_	1.24 ± 0.03	1.24 ± 0.04	1.22 ± 0.04	0.73 ± 0.08	0.79 ± 0.08	0.88 ± 0.04

A_T_: total alkalinity; *p*CO_2_: partial pressure of CO_2_ levels in seawater; [HCO_3_^-^]: bicarbonate ion concentration; [CO_3_^2−^]: carbonate ion concentration; Ω_calcite_, Ω_aragonite_: saturation states of the water with respect to calcite and aragonite minerals, respectively. All values correspond to mean ± standard error (n = 8).

### Physico-chemical parameters at the Quempillén estuary

The Quempillén estuary has been characterized as a shallow body water with a maximum depth of 3 meters and highly variable during the tidal and seasonal cycles. To characterize environmental conditions at the Quempillén estuary discrete measurements of the carbonate system parameters were performed along the sampling period (December 2016 to January 2018). Data of salinity and temperature were monthly obtained in situ using a CTD Idronaut (Ocean Seven 305 Plus). In addition, pH was determined using an espectophotometer Ocean View, following the method described by DOE [31], in which the light absorption caused by the weak acid and its conjugate base is measured through the use of the purple ink m-cresol [32]. All water samples were taken at 50 cm above bottom inhabited by the oyster bank. Values of temperature, pH, salinity and A_T_, were used to estimate the remaining parameters of the carbonate system and the saturation stage (Ω) of aragonite and calcite of the study site following the methodology describe above.

### Carbonate system parameter monitoring

Total scale pH (pH), total alkalinity (A_T_), salinity and temperature were measured in all 250 L tanks of the six treatments (i.e. 6 samples) once per week and at the same time in two randomly chosen experimental aquaria by treatment (i.e. 12 samples). The later carbon chemistry characterization (i.e. two aquaria by treatment) therefore incorporates the effects of the biota metabolism (two juvenile oysters by aquaria with the continuous supply of microalgae), temperature and manipulation on carbonate system speciation.

While pH and temperature were measured immediately after collection, A_T_ and salinity water samples were analyzed at the Centro de Investigación en Ecosistemas de la Patagonia (CIEP). Seawater inorganic carbon speciation was calculated from paired A_T_, pH, temperature and salinity values using the software CO2SYS [33] set with Mehrbach solubility constant [34] refitted by Dickson and Millero [35].

Total scale pH was measured at 25.0°C using a glass-fixed, ground-joint, diaphragm electrode with an integrated platinum resistance thermometer designed to work in low ion strange medium (model Aquatrode Plus from Metrohm®) previous one-point calibration using synthetic seawater Tris buffer [31]. We compared spectrophotometrically measured pH with the potentiometrically measured pH described above; founding absolute mean differences of 0.01 pH units in the salinity range from 22 to 27 (Alarcón, unpublished data). The agreement between both methods, confirmed that the potentiometric method was suitable to measure pH at the experimental salinity levels.

The total alkalinity (A_T_) was measured following the method of [36] using certified reference material supplied by Andrew Dickson (Scripps Institution of Oceanography) to verify A_T_ accuracy. Based on the analysis of blind A_T_ samples during “2017 Inter-laboratory Comparison of CO_2_ Measurements” (coordinated by Emily Bockmon and Andrew Dickson, unpublished data), we estimated deviations of approximately 0.1% from the reference value.

### Physiological measurements

All physiological rates were measured individually in five oysters for each combination of temperatures and *p*CO_2_ (six treatments) at the different experimental times (10, 20, 30 and 60 days).

#### Clearance Rate (CR)

Clearance rate (CR) was estimated in a static system, homogenised by aeration and using a food concentration of 75 x 10^6^ cells L^-1^ (2 mg dry weight L^-1^) of the microalgae *I*. *galbana*, which represent values of suspended particulate matter measured at the natural environment inhabited by *O*. *chilensis* [28]. The oysters were placed individually in 0.5 L glass chambers where the animals were exposed to the different *p*CO_2_ treatments and the corresponding experimental temperatures. The decrease in the number of cells was monitored every 30 min over a period of 4 h, using a particle counter (Beckman Z2) fitted with a 100 μm opening tube. The decrease in particle concentration in the experimental aquaria was maintained between 10 and 40% in relation to the initial concentration and was measured every 30 min for 4 h, with replacement of the consumed food. A control aquarium without oysters was used to estimate the sedimentation or the increment of particles during the experimental time. The CR (L h^−1^ oyster^−1^) was calculated according to Coughlan [37].

#### Absorption

Absorption efficiency (AE) was estimated using the ratio method of Conover [38]. Faeces were collected from each experimental oyster after clearance rate measurements were completed. Food and faeces samples were retained on pre-ashed, pre-weighed Whatman GF/C filters (1.2 μm pore size). These were then rinsed with ammonium formate (3%), dried to a constant weight at 100°C, weighed, combusted at 450°C for 3 h in a muffle furnace, and weighed again to determine the organic and inorganic fractions. Absorption rate was calculated as the product of the organic ingestion rate (clearance rate x organic content of the food) and absorption efficiency.

#### Oxygen uptake

Oxygen uptake was determined immediately after the CR measurements were completed (routine metabolism). Oysters were incubated in 140 mL glass sealed chambers filled with filtered seawater and oxygen uptake was measured using a Fiber Optic Oxygen Transmitter (FIBOX 3, PreSens) and oxygen sensor spots (PreSens GmbH, Regensburg, Germany) attached to the inner wall of the chambers. Oxygen sensors were previously calibrated in anoxic water using a saturated solution of Na_2_SO_3_ and in water 100% saturated with oxygen using bubbled air. The same chambers and experimental conditions, without animals, were used for controls. Data were recorded using the OxyView 3.51 software (PreSens GmbH). The glass chambers were placed in water baths at the different experimental temperatures (10, 15 and 20° C), controlled by Lauda RE112 equipments, and the dissolved oxygen was recorded. The oxygen concentration was not allowed to fall below 70% saturation.

#### Scope For Growth (SFG)

Measurements of the energy available for growth and reproduction, termed scope for growth, were made to provide a rapid and quantitative assessment of the energy status of the bivalve [39]. Scope for growth was calculated after converting the oxygen consumption rates and the organic matter from the diet to energy equivalents (J h^−1^):1 mL O_2_ = 19.9 J [40] and 1 mg of organic material from the diet = 21 J [41].

### Statistical analysis

A repeated-measures ANOVA were used to evaluate the temporal effects (i.e. 10, 20, 30, 60 days) of all physiological rates on *O*. *chilensis* individuals exposed to the three temperatures and two *p*CO_2_ levels. To meet ANOVA assumptions the data were transformed. The normality, homoscedasticity and sphericity of the data were tested using Kolmogorov-Smirnov, Levene's, and Mauchly's test, respectively. A post hoc Fisher test was used to determine the differences among the means of the physiological variables at different combinations of temperatures and *p*CO_2_ levels. Data were transformed (e.g. ln; square root) when they did not satisfy normality and homogeneity of variance. All analyses were carried out using STATISTICA 7.0 and differences were considered significant at p ≤ 0.05.

## Results

### Physico-chemical parameters at the Quempillén estuary

The environmental parameters measured during December 2016 to January 2018 show the seawater properties of the Quempillén estuary located in Southern Chile (Fig 1). High variability of temperature and salinity was observed throughout the sampling period, between 7.7 to 25.1°C and 7.4 to 32.6, respectively. The mean temperature of seawater was 14.7°C during the sampling period. Temperature reached a seasonal minimum of 7.7°C (June-July 2017). Seawater temperature returned to levels above 14°C by November 2017 and climbed to ~25°C by December 2017 (Fig 1A). During the autumn-winter season, cooling events and freshwater input were observed that coincided with periods of low pH levels and high *p*CO_2_ concentrations. The mean salinity was ~27, with the lowest values during the autumn-winter period (April to early-September). In addition, three abrupt decreased of salinity ranging between ~10–18 were registered between May and September and in January (Fig 1B). During December 2016 to early-May 2017 *p*CO_2_ levels remained under 400 μatm. However, there was an increase from a mean annual *p*CO_2_ concentration of 482 μatm to a single maximum value of 1646 μatm of *p*CO_2_ during spring (November 2017). Following this peak, *p*CO_2_ decreased rapidly during the summer period (Fig 1C). The pH during the summer months was maintained above ~7.8 pH but in the autumn-winter season decreased drastically to a minimum value of 6.97 (Fig 1D). Calcite and aragonite saturation states (Ω_cal_ and Ω_arg_) reflect the annual pattern of seawater pH at the Quempillén estuary. Results showed a mean value of 2.65 ± 0.18 for calcite and 1.68 ± 0.11 for aragonite (Fig 1E). The undersaturation levels of Ω_cal_ and Ω_arg_ were registered during the autumn-winter season (May to mid-September), and also during December 2017 (0.06 and 0.04 for Ω_cal_ and Ω_arg_, respectively). The maximum values of calcite and aragonite were registered during the spring-summer season (6.94 for Ω_cal_ and 4.49 for Ω_arg_), remaining saturated during this period of the year.

### Experimental conditions

The seawater conditions in which *Ostrea chilensis* were maintained during the experiments are summarized in Table 1. The pH_in situ_ (Total scale) varied slightly with the temperature within each level of *p*CO_2_, with average values of 7.89 and 7.69 at the low and high concentration of *p*CO_2_, respectively. The *p*CO_2_ showed higher variation with temperature at each *p*CO_2_ level, with a mean value of 504.73 μatm at the low *p*CO_2_ treatment and 850.86 μatm at the high *p*CO_2_ treatment. The carbonate concentration in the seawater decreased with increasing *p*CO_2_ levels whereas salinity (~27) and A_T_ (~1800 μmol Kg^-1^) remained similar between *p*CO_2_ and temperature treatments. Calcite value varies less between *p*CO_2_ and temperature treatments but under saturation of aragonite was reached under the high *p*CO_2_ treatment (i.e. 0.73, 0.79, 0.88) at 10, 15 and 20°C, respectively (Table 1).

### Physiological measurements

#### Clearance rate

**Fig 2:**
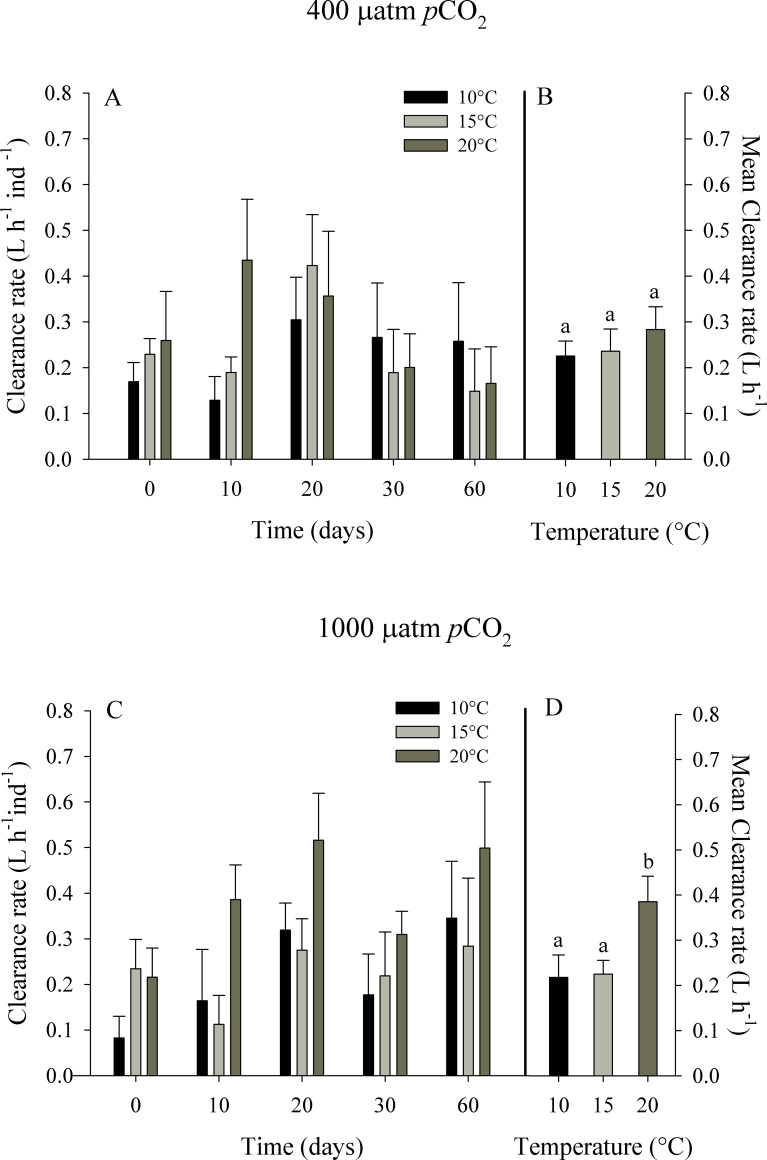
Clearance rate of juvenile *Ostrea chilensis* exposed to different combinations of temperature and *p*CO_2_ in relation to time. Clearance rate of oysters exposed to 400 μatm *p*CO_2_ (A) and to 1000 μatm *p*CO_2_ (C). Mean clearance rate of oysters exposed to 400 μatm *p*CO_2_ (B) and 1000 μatm *p*CO_2_ (D) during the whole experimental period. Values correspond to mean ± standard error of n = 25. Different letters indicate significant differences.

The clearance rate (CR) tended to increase as temperature increased (Fig 2). The mean CR of the control group (~400 μatm *p*CO_2_) varied between 0.23 ± 0.03 at 10°C and 0.28 ± 0.05 L h^-1^ ind^-1^ at 20°C. For the high level of *p*CO_2_ (~1000 μatm *p*CO_2_), the values oscillated between 0.22 ± 0.05 at 10°C and 0.39 ± 0.06 L h^-1^ ind^-1^ at 20°C. The analysis of variance of repeated measurements (RM-ANOVA) indicated a significant effect between levels of temperature (F_2,96_ = 3.947, p = 0.033) and time of exposure (F_4.96_ = 4.712, p = 0.002), but no significantly effects were found in relation of *p*CO_2_ levels on the clearance rate of *O*. *chilensis*. The interaction between *p*CO_2_ levels and temperature did not show significant effects (F_2,96_ = 1.031, p = 0.372).

#### Absorption

**Fig 3:**
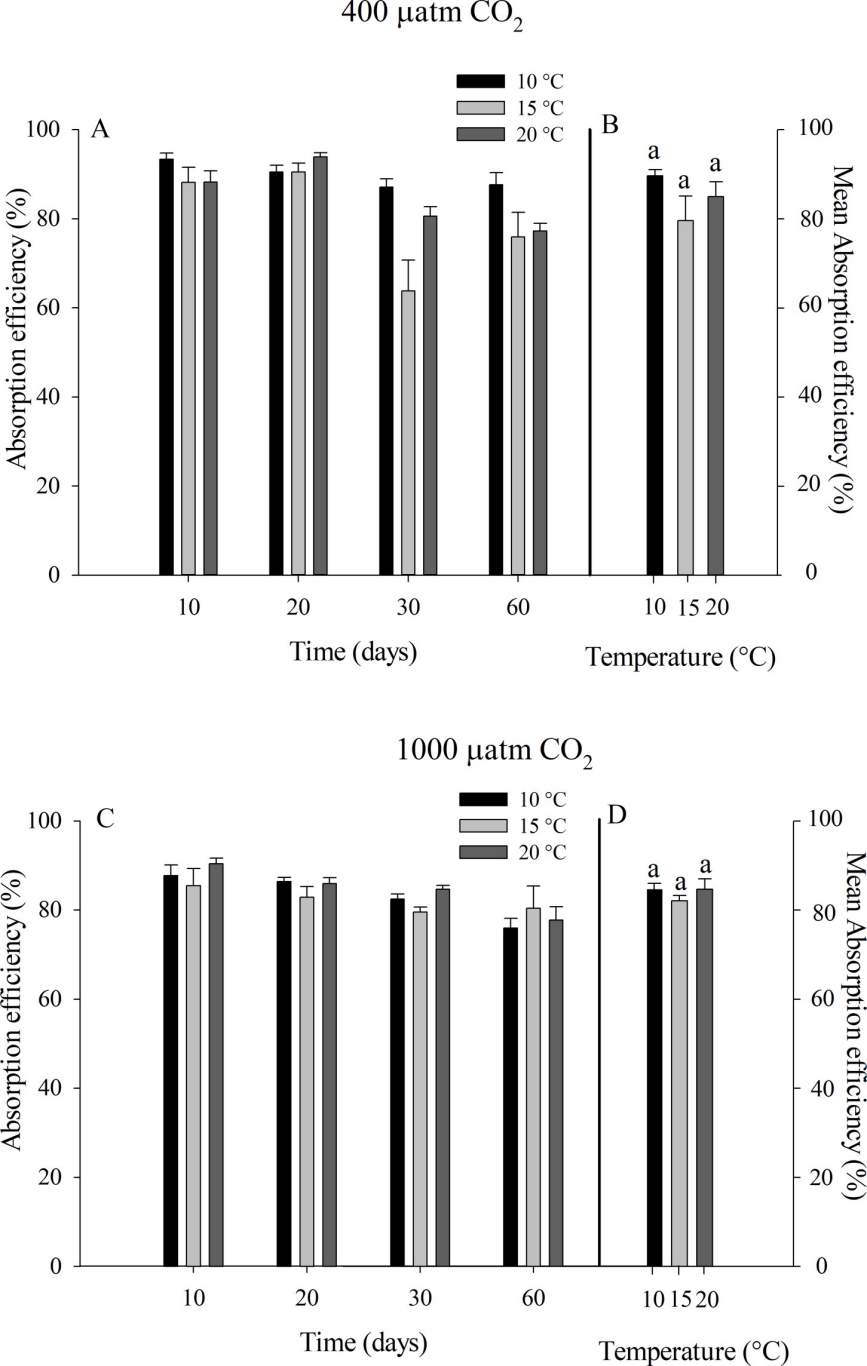
Absorption efficiency of juvenile *Ostrea chilensis* exposed to different combinations of temperature and *p*CO_2_ in relation to time. Absorption efficiency of oysters exposed to 400 μatm *p*CO_2_ (A) and to 1000 μatm *p*CO_2_ (C). Mean absorption efficiency of oysters exposed to 400 μatm *p*CO_2_ (B) and 1000 μatm *p*CO_2_ (D) during the whole experimental period. Values correspond to mean ± standard error of n = 25. Different letters indicate significant differences.

The absorption efficiency (AE) of *O*. *chilensis* did not show any pattern with respect to the variables studied. The mean values of AE for the control *p*CO_2_ treatment ranged from 79.60 ± 6.15% at 15°C and 89.62 ± 1.44% at 10°C and between 82.05 ± 1.33% at 15°C and 84.66 ± 2.63% at 20°C for the highest level of *p*CO_2_ (Fig 3). Non-significant differences were found between levels of temperature (10°C, 15°C and 20°C), and neither for high and low *p*CO_2_ treatments (400 and 1000 μatm of *p*CO_2_) and time of exposure in absorption efficiency of experimental oysters. Furthermore, non-significant temperature, *p*CO_2_ levels_,_ and time of exposure interaction was observed.

**Fig 4:**
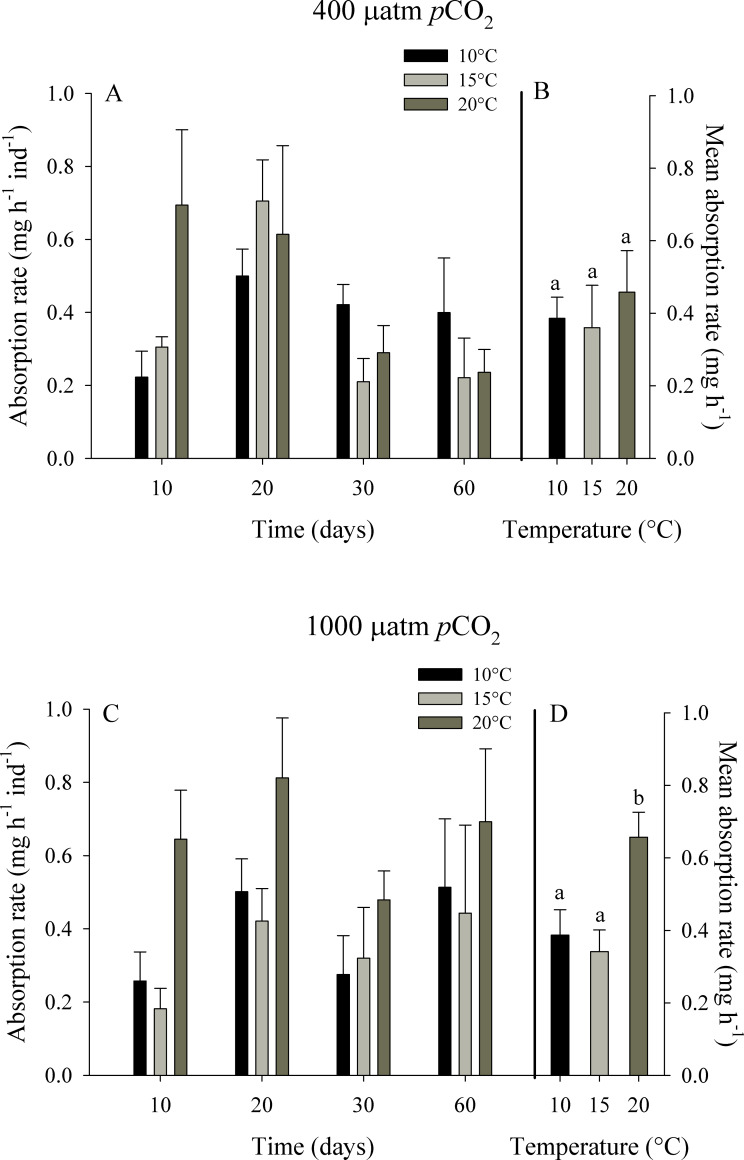
Absorption rate of juvenile *Ostrea chilensis* exposed to different combinations of temperature and *p*CO_2_ in relation to time. Absorption rate of oysters exposed to 400 μatm *p*CO_2_ (A) and to 1000 μatm *p*CO_2_ (C). Mean absorption rate of oysters exposed to 400 μatm *p*CO_2_ (B) and 1000 μatm *p*CO_2_ (D) during the whole experimental period. Values correspond to mean ± standard error of n = 25. Different letters indicate significant differences.

The absorption rate (AR) showed a tendency to increase with temperature, especially for the oysters exposed to ~1000 μatm *p*CO_2_ (Fig 4). Mean absorption rate fluctuated between 0.36 ± 0.12 at 15°C and 0.46 ± 0.11 mg h^-1^ at 20°C for the control *p*CO_2_ treatment, while for the high *p*CO_2_ treatment values ranged from 0.34 ± 0.08 at 15°C to 0.66 ± 0.07 mg h^-1^ at 20°C. The RM-ANOVA indicated significant effects of temperature (F_2,72_ = 4.311, p = 0.025) and time of exposure (F_3,72_ = 5.730, p = 0.001) on the absorption rate of *O*. *chilensis*.

No significant effect of *p*CO_2_ levels was observed, and the interaction between temperature, *p*CO_2_ levels and time of exposure was also not significant (F_6,72_ = 0.660, p = 0.682).

#### Oxygen uptake

**Fig 5:**
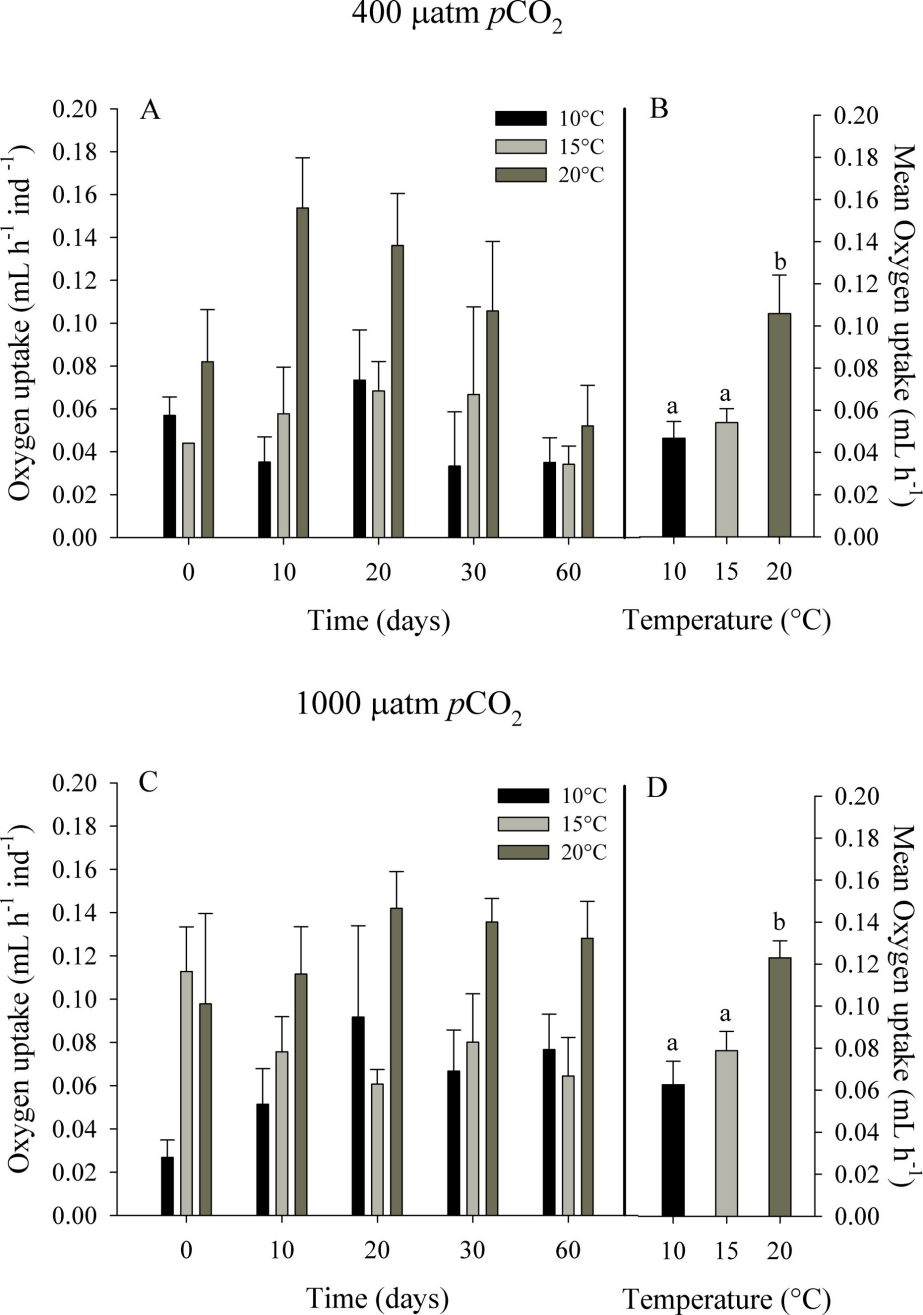
Oxygen uptake of juvenile *Ostrea chilensis* exposed to different combinations of temperature and *p*CO_2_ in relation to time. Oxygen uptake of oysters exposed to 400 μatm *p*CO_2_ (A) and to 1000 μatm *p*CO_2_ (C). Mean Oxygen uptake of oysters exposed to 400 μatm *p*CO_2_ (B) and 1000 μatm *p*CO_2_ (D) during the whole experimental period. Values correspond to mean ± standard error of n = 25. Different letters indicate significant differences.

The oxygen uptake (VO_2_) of *O*. *chilensis* showed a clear tendency to increase with an increase in temperature (Fig 5). Mean VO_2_ fluctuated between 0.05 ± 0.01 at 10°C and 0.11 ± 0.02 ml O_2_ h^-1^ at 20°C for the control treatment of *p*CO_2_. Similarly, values of VO_2_ of individuals exposed to high *p*CO_2_ levels (i.e. 1000 μatm of *p*CO_2_) ranged from 0.06 ± 0.01 at 10° C to 0.12 ± 0.01 ml O_2_ h^-1^ at 20°C. The RM-ANOVA indicated significant effects of temperature (F_2,96_ = 16.872, p = 0.000) and *p*CO_2_ level (F_1,96_ = 4.755; p = 0.039) on the oxygen uptake of *O*. *chilensis*, but the interaction of both factors was not significant (F_2,96_ = 0.111, p = 0.895). A significant effect of time of exposure (F_4,96_ = 2.879, p = 0.027) on oxygen consumption was also observed; however, no significant effects of the interaction of time of exposure with temperature and *p*CO_2_ level were observed (F_8,96_ = 1.939, p = 0.063).

#### Scope for growth

**Fig 6:**
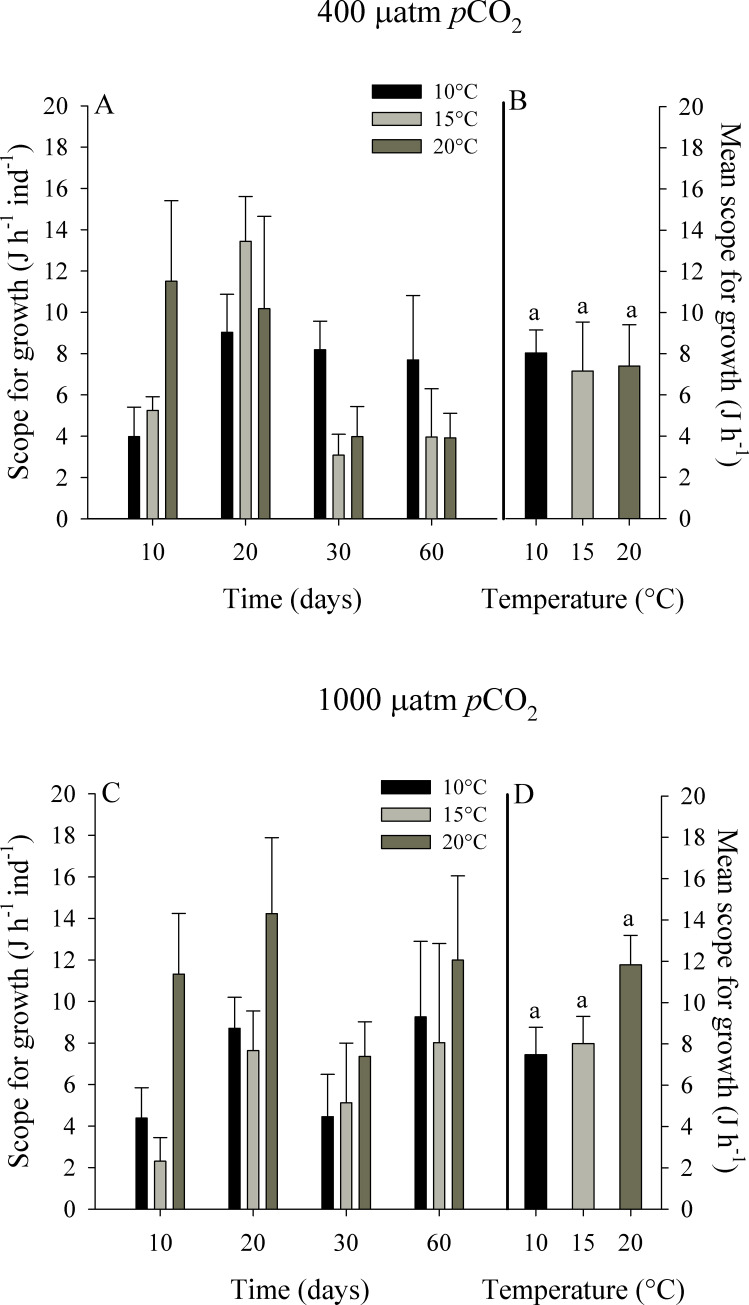
Scope for growth of juvenile *Ostrea chilensis* exposed to different combinations of temperature and *p*CO_2_ in relation to time. Scope for growth of oysters exposed to 400 μatm *p*CO_2_ (A) and to 1000 μatm *p*CO_2_ (C). Mean scope for growth of oysters exposed to 400 μatm *p*CO_2_ (B) and 1000 μatm *p*CO_2_ (D) during the whole experimental period. Values correspond to mean ± standard error of n = 25. Different letters indicate significant differences.

The scope for growth (SFG) of *O*. *chilensis* did not significantly differ between treatments of temperature and *p*CO_2_ level. In the control *p*CO_2_ treatment (i.e. ~400 μatm of *p*CO_2_) the mean values of SFG fluctuated between 6.43 ± 2.38 at 15°C and 7.40 ± 2.01 J h^-1^ at 20°C, while for the high *p*CO_2_ treatment (i.e. ~1000 μatm of *p*CO_2_), SFG varied between 5.77 ± 1.32 J h^-1^ at 15°C and 11.22 ± 1.43 J h^-1^ at 20°C (Fig 6). The RM-ANOVA indicated that only time of exposure had a significant effect on the scope for growth of *O*. *chilensis* (F_3,72_ = 5.140, p = 0.003). Despite this, no significant effects of the interaction of time of exposure with temperature and *p*CO_2_ level were observed (F_6,72_ = 0.765, p = 0.600).

## Discussion

This is one of the first studies to report natural patterns of seawater such as A_T_, pH, temperature, salinity and associated carbonate system chemistry along the year in an estuary of Southern Chile. The monitoring carried out from December 2016 to January 2018 at the Quempillén estuary add an additional information to our general understanding of seasonal variability in carbonate chemistry of the mid latitude estuarine systems. The values of temperature and salinity registered at the Quempillén estuary are agreeing with previous studies [28] for the same study area, where these authors registered that temperature reaches a maximum of 20°C during low summer tide and a minimum of 8.6°C at low winter tide. Similarly, salinity varies considerably depending on the tide, and variability in salinity is especially notable in winter with extreme values ranging from 9.4 to 30.3 at low and high tide, respectively [28]. Despite the importance of alteration in carbonate chemistry and pH of seawater in coastal and estuarine systems, the annual variation in pH and saturation state of calcite and aragonite has been scarce studied in estuaries of Southern Chile [42]. Several studies have demonstrated that reduction in carbonate ion concentration can affect the ability of marine organisms to produce and/or maintain calcium carbonate (CaCO_3_) calcareous structures [1]. Based on our data, the undersaturation levels of Ω_cal_ and Ω_arg_ were registered during autumn-winter season and relatively saturated conditions were registered during spring-summer season. Similarly, Vergara-Jara et al. [43] shown strong seasonal variability of pH and *p*CO_2_ in the Reloncaví fjord, Chilean Patagonia, with the undersaturation levels of Ω_arg_ in winter and saturated conditions of aragonite in the summer. Our study shows the first time series of temperature and salinity associated with the parameters of the carbonate system within the Chilean oyster farm. The environmental conditions described for the Quempillén estuary share similarities with other estuaries in the region, such as a marked seasonal cycle characterized by minimum values of temperature and pH during the winter [43]. However, the intensity and timing of freshwater input to the estuary, as well as its concomitant effects on salinity and speciation of carbon chemistry, are expected to vary in association with local hydrographic characteristics (eg. glacial influence / precipitation, basin geomorphology and lithology) [44] and to the variations imposed by climate change (disturbances in the hydrological cycle), therefore modulating the characteristics of the ocean acidification projected in the open sea for the Patagonian zone [45]. The combined effects of experimental temperature and *p*CO_2_ did not affected the physiological rates of the oyster *O*. *chilensis*. However, it is important to emphasize that non-negative effects of temperature, *p*CO_2_ and/or their interaction on individuals of *O*. *chilensis*, not necessary preclude that other physiological changes can occur (e.g. calcification and dissolution rates). Our findings highlight the physico-chemical complexity (i.e. environmental parameters along the year) of estuarine systems and the importance such data hold for the design of ecologically relevant low pH/high *p*CO_2_ experiments as well as experiments in which multiple stressors are included.

Estuarine organisms are described as more tolerant to environmental fluctuations than their fully coastal and oceanic counterparts which inhabit at relatively more stable environments [46]. Oysters belong to one of the groups of calcifying organisms inhabiting estuaries and coastal areas, environments that could be strongly affected by ocean acidification and other drivers of climate change [47]. Higher clearance rates for the oyster *Pinctada fucata* were reported when individuals were exposed to acidic conditions [48]. According to Pörtner and Farrel [10], ocean acidification may narrow the thermal tolerance window of aquatic animals, possibly due to the accumulation of CO_2_ in tissues that reduce their functional capacity. The lack of lethal or sublethal effects of high *p*CO_2_ and high temperature on *O*. *chilensis* could be explained by the environmental conditions experienced by this species at the Quempillén estuary, where temperature fluctuates between 7.7 and 25 ºC and pH varies between 8.6 and 7.0 throughout the year. Although the extreme values of temperature and *p*CO_2_ at the Quempillén estuary occur only for a short period of time (hours) during the year, the present study represents more the plastic response of *O*. *chilensis* to the temperature and *p*CO_2_ within the present natural range of variability, than the response to climate change. Estuaries and fjords are places where high concentrations of *p*CO_2_ can be naturally observed. The *p*CO_2_ concentration of the Kiel fjord (Western Baltic Sea) peaks to 2400 μatm (pH 7.4) in spring and summer, yet still several calcifying invertebrates maintain high levels of recruitment during these peaks [49]. The same authors have also shown that somatic and shell growth of juvenile *Mytilus edulis* occurs at 1400 μatm of *p*CO_2_ (pH 7.6). Therefore, the contrasting responses of marine organisms to environmental stressors (for example, high levels of *p*CO_2_ and temperature) can be understood as local adaptation to the high variability of the coastal environments [50].

The AE of *O*. *chilensis* was not affected by temperature and *p*CO_2_, nor by the interaction of these variables. Similar studies, with other species of bivalves have also shown that temperature and *p*CO_2_ do not affect absorption efficiency [51,52]. Zhang et al. [53] found that AE of the gastropod *Nassarius conoidalis* was not significantly affected at the beginning of the experimental period (day 2) by temperature, *p*CO_2_ level, nor the interaction between both factors. However, on day 30, the temperature negatively affected AE when this gastropod was exposed at the highest *p*CO_2_ concentration. Thus, medium and long-term experiments avoid misinterpretations due to the initial acclimation ability of a species to different environmental stressors. Fernández-Reiriz et al. [54] have shown that the absorption efficiency of *Mytilus galloprovincialis* is low when individuals are exposed to high *p*CO_2_/low pH conditions, (e.g. similar to Navarro et al. [22] for *Mytilus chilensis*). Similarly, to our study, Zhang et al. [53] showed that absorption rate of the gastropod *Nassarius conoidalis* is highest when was exposed to both high *p*CO_2_ and high temperature (30 ºC). These results can be explained because the effect of warming will depend on the thermal window of the species under study. In the present work we used oysters collected from the estuary of Quempillén, with temperature ranging between 7 and 25 ºC. In general, these studies suggest that AE responses of organisms to different drivers of climate change can be species-specific, or in some cases population-specific.

The standard metabolic rate of *Crassostrea virginica* is higher under acidified conditions, which was attributed to the higher energy costs [7]. Christensen et al. [55] described the combined effects of pH and temperature on the ophiuroid *Ophionereis schayeri* exposed to acidified treatments, showing that this species consumes more oxygen at low pH and high temperature conditions. Similarly, in our study, the oxygen uptake of *Ostrea chilensis* was higher with higher temperature and *p*CO_2_. Despite this, these higher metabolic rates observed at 20 ºC were supported by high values of energy intake (i.e. clearance and absorption rates); which represent a physiological trade-off to maintain positive scope for growth, with values not significantly different between treatments. The highest mean value of scope for growth was found for the 20 ºC and the high *p*CO_2_ treatment. High scope for growth values have been also found for the giant mussel *Choromytilus chorus* subjected to acidic conditions [51]. *Ostrea edulis* exposed to a wide range of temperature (14–26°C) showed the higher ingestion rates and scope for growth values at the highest temperatures [56]. However, the positive effects of temperature occur when an organism is exposed to temperatures within its range of thermal tolerance; but experiencing more acute conditions compromises the energy needed to regulate physiological responses. On the other hand, Hiebenthal et al. [57] show that combined temperature/*p*CO_2_ treatments are associated with increased mortality, cellular stress, and reduced growth in *Mytilus edulis*. As such, it has been proposed that temperature increase could positively affect metabolism, partially counteracting the negative effects of acidification [22–58]. Thus, the responses of different species are highly variable and species-specific when the impacts of high *p*CO_2_ levels are assessed in combination with temperature increased [59,60].

Marine organisms, particularly those inhabiting shallow waters, survive short-term exposure either to increased temperature or to increased acidification. However, several studies report increased mortality given increasing exposure time [14–61]. Here we verify the importance of the factor time since this had a significant effect on all the physiological variables measured. Thus, *Ostrea chilensis* inhabiting at the Quempillén estuary showed a great physiological plasticity to live in this environment, where extreme environmental conditions (e.g. 25 ºC and 1650 μatm of *p*CO_2_) occur only in short periods of time (few hours), where the bivalves are able to close their valves and isolate themselves, until suitable conditions occur again.

Different responses to high *p*CO_2_ conditions have been identified for populations from different geographic locations. From this it has been indicated that phenotypic plasticity allows organisms to adapt to environmental fluctuations [62,63]. Therefore, the response of species to climate change scenarios will strongly depend on the environmental variability that organisms experience in their natural habitats [50–64]. To avoid extinction in the face of climate change, it is expected that marine organisms will have to modify their range of geographical distributions, have adequate physiological plasticity, or have the ability to adapt genetically. In this study, *Ostrea chilensis* showed high plasticity to respond to the high levels of temperature and *p*CO_2_ experienced in its habitat as no negative physiological effects were observed within this 60-day exposure period. Thus, the highly variable conditions of this organism’s environment could select for individuals that are more resistant to future scenarios of climate change, mainly to warming and acidification. Although the ranges of temperature and *p*CO_2_ studied here do not affect negatively the physiological rates of juveniles *O*. *chilensis*, these environmental stressors have shown affect calcification and shell properties (e.g. biomineralization and biomechanical characteristics) of several bivalve species [65,66]. Thus, the combined effects of these stressors and other environmental factors with levels predicted for future scenarios of climate change should be considered in future research.

## Supporting information

S1 Data (XLSX)
